# Identification of a Novel Chromate and Selenite Reductase FesR in *Alishewanella* sp. WH16-1

**DOI:** 10.3389/fmicb.2022.834293

**Published:** 2022-03-08

**Authors:** Zijie Zhou, Lin Zhu, Yixuan Dong, Lexing You, Shixue Zheng, Gejiao Wang, Xian Xia

**Affiliations:** ^1^State Key Laboratory of Agricultural Microbiology, College of Life Science and Technology, Huazhong Agricultural University, Wuhan, China; ^2^College of Geography and Environmental Sciences, Zhejiang Normal University, Jinhua, China; ^3^Hubei Key Laboratory of Edible Wild Plants Conservation & Utilization, Huangshi Key Laboratory of Lake Environmental Protection and Sustainable Utilization of Resources, Hubei Engineering Research Center of Characteristic Wild Vegetable Breeding and Comprehensive Utilization Technology, College of Life Sciences, Hubei Normal University, Huangshi, China

**Keywords:** *Alishewanella*, FesR, chromate reduction, selenite reduction, electron transport

## Abstract

A ferredoxin protein (AAY72_06850, named FesR) was identified to associate with chromate [Cr(VI)] resistance in *Alishewanella* sp. WH16-1. FesR and its similar proteins were phylogenetically separated from other reductase families. Unlike the reported Cr(VI) and selenite [Se(IV)] reductases, two 4Fe-4S clusters and one flavin adenine dinucleotide (FAD) -binding domain were found in the FesR sequence. The experiment *in vivo* showed that the mutant strain Δ*fesR* had lost partial Cr(VI) and Se(IV) reduction capacities compared to the wild-type and complemented strains. Furthermore, overexpression in *Escherichia coli* and enzymatic tests *in vitro* showed FesR were involved in Cr(VI) and Se(IV) reduction. 4Fe-4S cluster in purified FesR was detected by ultraviolet-visible spectrum (UV-VIS) and Electron Paramagnetic Resonance (EPR). The Km values of FesR for Cr(VI) and Se(IV) reduction were 1682.0 ± 126.2 and 1164.0 ± 89.4 μmol/L, and the Vmax values for Cr(VI) and Se(IV) reduction were 4.1 ± 0.1 and 9.4 ± 0.3 μmol min^–1^ mg^–1^, respectively. Additionally, site-directed mutagenesis and redox potential analyses showed that 4Fe-4S clusters were essential to FesR, and FAD could enhance the enzyme efficiencies of FesR as intracellular electron transporters. To the best of our knowledge, FesR is a novel Cr(VI) and Se(IV) reductase.

## Introduction

The transition metal chromium (Cr) is widely used in industry (Lunk, 2015; Xia et al., 2021). The wide applications and long mining history have led to Cr contamination. Cr(VI) (chromate) and Cr(III) are the most common and stable forms in the natural environment (Xia et al., 2021). Cr(VI) is highly toxic because its easy migrates, while Cr(III) has low toxicity as low solubility and easy immobilization (Moukarzel, 2009; Wang et al., 2017). Selenium (Se) is an essential trace element for human health, but excessive intake may increase the risk of type-2 diabetes and other diseases (Rayman, 2012). There are four valences of Se in the natural environment: Se(IV), Se(IV) (selenite), Se(0) and Se(-II). Se(IV) is highly toxic and soluble, while Se(0) is non-toxic as low solubility (Wang et al., 2022). Accordingly, Cr(VI) and Se(IV) reduction is a very important way to remediate Cr and Se contamination. Various microbes have developed high reduction capacities to cope with Cr(VI) and Se(IV) (Wang et al., 2022; Xia et al., 2021). The application of these microorganisms in the bioremediation of Cr and Se contamination takes advantage of the low cost and the lack of secondary pollution (Jobby et al., 2018). However, the mechanisms of Cr(VI) and Se(IV) reduction in the microorganism remain to be further studied.

Cr(VI) and Se(IV) reduction mechanisms in the microorganism can be divided into enzymatic and non-enzymatic reduction (Thatoi et al., 2014; Xia et al., 2021). Non-enzymatic reduction is mediated by reducing substances such as GSH, sulfide, ascorbic acid, iron siderophore, and FADH_2_ (Thatoi et al., 2014; Wang et al., 2022). Thus far, several Cr(VI) and Se(IV) reductases have been identified in the bacteria. Under anaerobic conditions, Cr(VI) enzymatic reduction is coupled with respiratory chains. In *Shewanella oneidensis* MR-1, Cr(VI) can accept electrons from outer-membrane cytochromes MtrC and OmcA (Belchik et al., 2011). Under aerobic conditions, flavoproteins such as ChrR (Ackerley et al., 2004b), YieF (Ackerley et al., 2004b), NfsA (Ackerley et al., 2004a), NfoR (Han et al., 2021), NemA (Robins et al., 2013) and OYE family protein (Opperman et al., 2008) are responsible for Cr(VI) reduction. For anaerobic Se(IV) reduction, some reductases, such as sulfite reductase, nitrite reductase, hydrogenase I, arsenate reductase, fumarate reductase, and Srr complex (Wang et al., 2022), are produced by bacteria. For aerobic Se(IV) reduction, two reductases, SerT (Tan et al., 2018) and GorA (Wang et al., 2019), have been previously identified by our group in different microbes. Usually, these reductases use FMN, flavin adenine dinucleotide (FAD), cytochrome, Fe-S cluster, or Mo as cofactor (Xia et al., 2021; Wang et al., 2022). However, no bacterial Cr(VI) and Se(IV) reductases containing both Fe-S cluster and FAD have been deeply studied.

Flavin adenine dinucleotide (FAD) plays an important role in various biological processes, such as electron transport, nucleotide biosynthesis, DNA repair, amino acid catabolism, and beta-oxidation of fatty acids (Joosten and van Berkel, 2007). In terms of electron transport, the dissociative FAD can act as an extracellular electron shuttle (EES) to couple with the respiratory chain to enhance extracellular reduction under anaerobic conditions (Kotloski and Gralnick, 2013; Glasser et al., 2017). Flavin adenine dinucleotide can also non-covalently bind to protein to participate in redox reactions; for example, glutathione reductase GorA can reduce Se(IV) in *Pseudomonas stutzeri* TS44 under aerobic conditions (Wang et al., 2019). In addition, the reduced FAD (FADH_2_) can act as Cr(VI) reduction agent in the intracellular (Cervantes et al., 2001). However, the role of FAD plays in intracellular electron transport still needs to be further studied.

Previously, we isolated a high Cr(VI) and Se(IV) resistance and reduction strain, *Alishewanella* sp. WH16-1 (Zhou et al., 2016) and constructed a Tn*5* transposon mutagenesis library based on Cr(VI) resistance. Later, several genes responsible for Cr(VI) and Se(IV) resistance and reduction of WH16-1 have been identified, including chromate transporter ChrA (Xia et al., 2016, 2018a), terminal respiratory oxidase Cytbd (Xia et al., 2018a), superoxide ChrC (Xia et al., 2016), Cr(VI) and Se(IV) reduction flavoenzyme CsrF (Xia et al., 2018b) and DNA repairing system RuvRCAB (Wu et al., 2019). A potential novel reductase FesR-coding gene was identified by Tn*5* transposon mutagenesis, and the accession number of FesR was AAY72_06850. In this study, we comprehensively analyzed the Cr(VI) and Se(IV) reduction mechanism of FesR.

## Materials and Methods

### Bacterial Strains and Growth Condition

The strains and plasmids used in this study are listed in Supplementary Table 1, and the primers are listed in Supplementary Table 2. *Alishewanella* sp. WH16-1 and *Escherichia coli* strains S17-1 and BL21(DE3) were cultured at 37°C in Luria-Bertani (LB) medium. Stock solutions of rifampin (Rif, 50 mg mL^–1^), kanamycin (Kan, 50 mg mL^–1^), chloramphenicol (Cm, 25 mg mL^–1^), ampicillin (Amp, 50 mg/mL), K_2_CrO_4_ (1 mol/L) and Na_2_SeO_3_ (1 mol/L) were added when required. Rif, Kan, Cm, K_2_CrO_4_, and Na_2_SeO_3_ were obtained from Sinopharm Chemical Reagent Co., Ltd (Shanghai, China).

### Sequence Analysis

The amino acid sequence of FesR was first blasted against the NCBI Conserved Domains Database. Next, the predicted Fe-S cluster and FAD-binding conserved domain sequences were analyzed in the HHpred server (Alva et al., 2016). The similar sequences were downloaded and aligned with FesR conserved domain sequences using ClustalW (Thompson et al., 1994) and Espript 3.0 (Robert and Gouet, 2014). The transmembrane motif and signal peptide were predicted by TMHMM v. 2.0 (Krogh et al., 2001) and SignalP 5.0 (Almagro Armenteros et al., 2019), respectively. In addition, FesR was aligned with the reported chromate reductase by MEGA 7.0 (Kumar et al., 2016), and a phylogenetic tree was constructed by FigTree V1.4.3.^1^

### Construction of Complementary and Overexpressed Strains

For complementary strain construction, the *fesR* gene and its promoter region were amplified by C-*fesR*F/C-*fesR*R (Supplementary Table 2) and then cloned into the plasmid pCT-Zori by the restriction endonucleases *Hin*dIII/*Sac*I. The generated pCT-Zori-*fesR* vector was verified by sequencing and was then transformed into *E. coli* S17-1. Next, *E. coli* S17-1-pCT-Zori-*fesR* was conjugated with Δ*fesR* to obtain a complementation strain Δ*fesR*-C by Rif and Cm resistance selection. The complementary strain was confirmed by PCR using the primers V-*fesR*F/V-*fesR*R (Supplementary Table 2) and sequenced.

To construct the overexpression strain, the *fesR* gene and its promoter region ERE were amplified by pfu (TransGen), and an A tag was added to the generated fragment by rTaq (Takara). Then, the fragment was ligated to the plasmid pGEM-T by T4 ligase. The resulting plasmid pGEM-T-*fesR* was confirmed by PCR and sequencing. The constructed plasmid was transformed into *E. coli* S17-1 to obtain the overexpression strain *E. coli* S17-1-T-*fesR*.

### Growth and Se(IV) or Cr(VI) Reduction Experiments

Strains WH16-1, Δ*fesR*, and Δ*fesR*-C were added in a 250 mL conical flask containing 100 mL of LB and incubated at 37°C with shaking at 150 rpm. When the OD_600_ reached 0.8–1.0, K_2_CrO_4_ or Na_2_SeO_3_ stock solutions were added with the final concentrations of 1000 and 500 μmol/L, respectively. Two-milliliter samples were collected at the designated time, one milliliter for growth determination, and the other for determination of the residual Se(IV) or Cr(VI) concentration. The growth curves for Cr(VI) and Se(IV) reduction were detected by UV-VIS (OD600nm) spectroscopy (UV1900, Shanghai AOE Instruments Co., Ltd, China) and Coomassie Brilliant Blue G250 staining to measure growth based on the cellular protein content (Xia et al., 2018b). To measure the residual Se(IV), the samples were centrifugated (12,000 rpm for 5 min) to remove the Se(0) produced by Se(IV) reduction (Xia et al., 2018b). The residual Se(IV), which existed in the supernatant, was then measured by High Performance Liquid Chromatography-Atomic Fluorescence Spectrometer (HPLC-AFS) (Xia et al., 2018b).Cr(VI) was measured by colorimetric diphenylcarbazide (DPC) methods (Xia et al., 2018b).

In addition, the *fesR* sequence was obtained by the primers pGEX*-fesR*F/pGEX*-fesR*R. Then, the DNA fragment was inserted into pEGM-T by TA-cloning, constructing pEGM-T-*fesR*. To obtain *E. coli* S17-1-T-*fesR* and *E. coli* S17-1-T by transforming pEGM-T-*fesR* and empty pEGM-T into *E. coli* S17-1, respectively. *E. coli* S17-1, *E. coli* S17-1-T, and the overexpression strain *E. coli* S17-1-T-*fesR* were also used to test growth and reduction. To avoid the K_2_CrO_4_ or Na_2_SeO_3_ inhibiting the early growth of *E. coli* S17-1, *E. coli* S17-1-T, and *E. coli* S17-1-T-*fesR*, all of these strains were cultured to OD_600_ values of approximately 1.0 and were then supplemented with K_2_CrO_4_ or Na_2_SeO_3_ stock solutions to final concentrations of 500 μmol/L. The growth and reduction capacities were tested as described above.

### Heterologous Expression and Purification of FesR

The target FesR was obtained from *E. coli* BL21(DE3) by heterologous expression. The *fesR* sequence was amplified by the primers pGEX*-fesR*F/pGEX*-fesR*R. Then, the DNA fragment was digested by *Eco*RI/*Xho*I and cloned to pGEX-6p-1, generating pGEX-6p-1-*fesR*. The vector was transformed into *E. coli* BL21(DE3) after sequence confirmation. The heterologous expression strain *E. coli* BL21(DE3)-pGEX-6p-1-fesR was grown at 37°C until an OD_600_ of 0.3–0.4. Then, 1000-mL cultures were incubated with 0.2 mmol/L IPTG for 4–6 h at 28°C. Next, cells were collected and resuspended in Tris-HCl buffer (pH 7.5). To obtain the crude FesR, the collected cells were lysed using a low-temperature ultra-high pressure continuous flow cell disrupter (JN-3000PLUS, Juneng Nano & Bio-Technology Co., Ltd., Guangzhou, China) at 100 MPa.

The purification of FesR was carried out by affinity column chromatography. The crude FesR flowed through the affinity column (GST), and 35 mL Tris-HCl buffer (pH 7.5) and 6 mL Tris-HCl buffer (pH 8.0) with 10 mmol/L reduced glutathione were then used to clean miscellaneous proteins in the affinity column (GST). To elute FesR from the affinity column (GST), 8 mL of Tris-HCl buffer (pH 8.0) with 10 mmol/L reduced glutathione was used. The purified FesR was centrifuged in a microsep and then dissolved in 200 μL of Tris-HCl buffer (pH 8.0). The quality and quantity were detected by SDS-PAGE and spectrophotometry (NanoDrop 2000, Thermo), respectively.

### Analysis of the Enzymatic Characteristics of FesR

The properties of FesR were studied by evaluating catalytic parameters such as kinetics and, optimal pH and temperature for the reduction of Cr(VI) and Se(IV). FesR was used to reduce Cr(VI) and Se(IV) by different reaction systems. For determination of the optimum pH, 10 μg/mL FesR was incubated with 1 mmol/L NADPH and 1 mmol/L K_2_CrO_4_ for 10 min or Na_2_SeO_3_ in 50 mmol/L Tris-HCl (pH 3–11) for 30 min in a reaction volume of 100 μL. A similar system was used to detect the optimum temperature in the range of 10–80°C. For research of the influence of different electron donors (NADH or NADPH) on the enzyme activity of FesR, 10 μg/mL FesR was incubated with 1 mmol/L electron donor (NADH or NADPH) and 0, 200, 400, 600, 800, 1000, 1500, 2000, 3000 and 4000 μmol/L K_2_CrO_4_ for 10 min at optimum pH and temperature in a reaction volume of 100 μL. To obtain the preference of reduction of FesR where equimolar Cr(VI) and Se(IV) were added together, 10 μg/mL FesR was incubated with 1 mmol/L NADPH and 1 mmol/L of equimolar K_2_CrO_4_ and Na_2_SeO_3_ for 10 min at optimum pH and temperature in a reaction volume of 100 μL. For kinetics studies, 10 μg/mL FesR was incubated with 1 mmol/L NADPH and 0, 200, 400, 600, 800, 1000, 1500, 2000, 3000 and 4000 μmol/L K_2_CrO_4_ for 10 min or 0, 300, 500, 700, 1000, 1500, 2000, 3000 and 4000 μmol/L Na_2_SeO_3_ for 10 min at optimum pH and temperature in a reaction volume of 100 μL. At the designated time, the samples were heated (100°C for 10 min) to stop the enzymatic reaction and then harvested by centrifugation (12,000 rpm for 5 min) to collect the supernatant. The remaining concentration of Cr(VI) and Se(IV) in the supernatant was obtained by DPC and HPLC-AFS method, respectively (Xia et al., 2018b). The enzyme activity was described by the consumption of Cr(VI) and Se(IV) in unit reaction time (Scopes, 2002). The characteristics were analyzed by GraphPad Prism software version 5.01 (GraphPad Software, San Diego, California, United States).

### Site-Directed Mutagenesis

According to the sequence analysis results, the key amino acid residues (Arg385, Cys541, Cys544, and Cys598) involved in FAD and 4Fe-4S binding were mutated. Site-directed mutagenesis was performed by the same method of our previous study (Xia et al., 2018b). The plasmid pGEX-6p-1-*fesR* was used as a PCR template, and the site-directed mutagenesis primers are shown in Supplementary Table 2. The mutant plasmids were transformed into *E. coli* BL21(DE3) to express the mutant protein M385(Arg385 was mutated into Ala385), M541(Cys541 was mutated into Ala541), M544(Cys544 was mutated into Ala544), and M598(Cys598 was mutated into Ala598) after sequencing confirmation. In order to obtain the 8 cysteines (Cys538, Cys541, Cys544, Cys548, Cys549, Cys595, Cys598, Cys601, and Cys605) mutant MFesR, the pGEX-6p-1-m*fesR* plasmid (the nucleotide acids of cysteine were replaced by the nucleotide acids of alanine) was synthesized by Tsingke Biotechnology Co., Ltd. The mutants and wild-type FesR were purified. All mutant proteins (20 μg/mL) were incubated with 400 μmol/L NADPH and 1 mmol/L K_2_CrO_4_ for 30 min or Na_2_SeO_3_ for 120 min at optimum pH and temperature in a reaction volume of 100 μL. In addition, wild-type FesR and M385 were incubated with 400 μmol/L NADPH, 300 μmol/L FAD, and 1 mmol/L K_2_CrO_4_ for 30 min or Na_2_SeO_3_ for 120 min at optimum pH and temperature in a reaction volume of 100 μL. The residual amounts of Cr(VI) and Se(IV) were measured as described above.

### Ultraviolet-Visible Spectrum Spectrum and Electron Paramagnetic Resonance Analysis

The UV-VIS spectrum of FesR (wild-type protein), FeS (only contain 4Fe-4S domain), MFesR (4Fe-4S cluster binding domains were mutant) were obtained by UV-1900 spectrophotometer (AoYi Instruments Shanghai Co. Ltd) at room temperature. EPR was obtained by an A300 EPR spectrometer (Bruker Beijing Scientific Technology Co. Ltd). EPR was performed under microwave frequency, 9.85 GHz; microwave power, 19.15 mW; modulation frequency, 100 kHz; modulation amplitude, 1G; receive gain, 1 × 10^3^.

### Electrochemical Characterization

The redox potentials of FAD and FesR were measured in a three-electrode system. A saturated calomel electrode (SCE) was used as the reference electrode, and a Pt wire served as the counter electrode. Before the experiments, bare glassy carbon (GC) working electrodes 3 mm in diameter were in turn polished with 1, 0.3, and 0.05 μm Al_2_O_3_ powder and rinsed with ultrapure water three times. The electrodes were then transferred into 0.1 M phosphate buffer solution containing 1 mM K_3_[Fe(CN)_6_] (pH = 7) to check their cleanliness. The redox potentials of FAD and FesR were subsequently measured with clean GC electrodes in 50 mM Tris-HCl buffer solution (pH = 8) by using differential pulse voltammetry (DPV) (CHI660E, Shanghai Chenhua Instruments Co., Ltd., China). Nitrogen was bubbled into the buffer solution during the entire experiment at room temperature (25°C).

## Results

### The Conserved Domain of FesR Differed From Those of Reported Cr(VI) and Se(IV) Reductases

**FIGURE 1:**
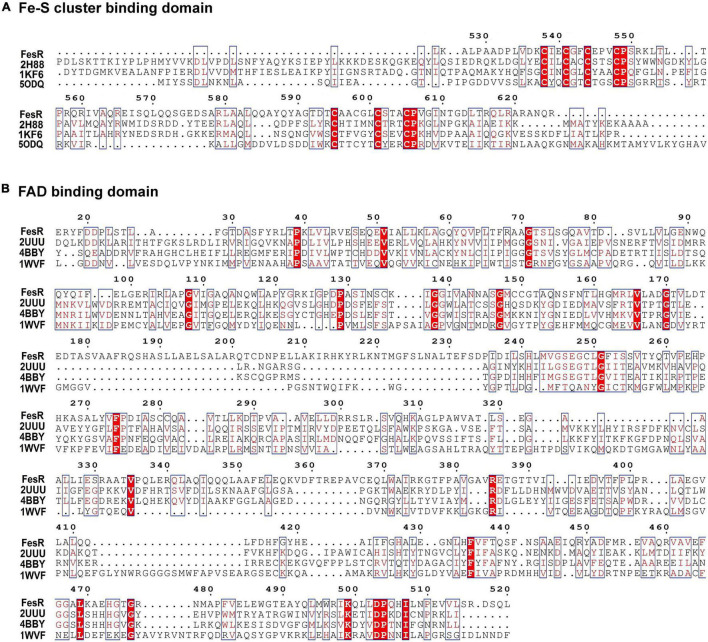
Multiple sequence alignment of flavin adenine dinucleotide (FAD) **(A)** and Fe-S cluster **(B)** conserved binding domains. Predicted FAD (16–520 aa)- and 4Fe-4S cluster (525–625 aa)-binding sites were used to search in the HHpred server. The results showed that FesR shared high similarity in the FAD-binding domain with 2UUU, 4BBY, and 1WVF and high similarity in the 4Fe-4S cluster-binding domain with 2H88, 1KF6, and 5ODQ. These proteins were downloaded from the PROTEIN DATA BANK (PDB) and were used for further sequence alignment. Identical residues are shown in red background, and highly similar residues are shown in the red boxes. Arg385 might be involved in FAD binding, while Cys538, Cys541, Cys544, and Cys548 might be responsible for 4Fe-4S binding and Cys595, Cys598, Cys601, and Cys605 responsible for another 4Fe-4S binding according to the sequence analysis results and the literature (Landry and Ding, 2014).

**FIGURE 2:**
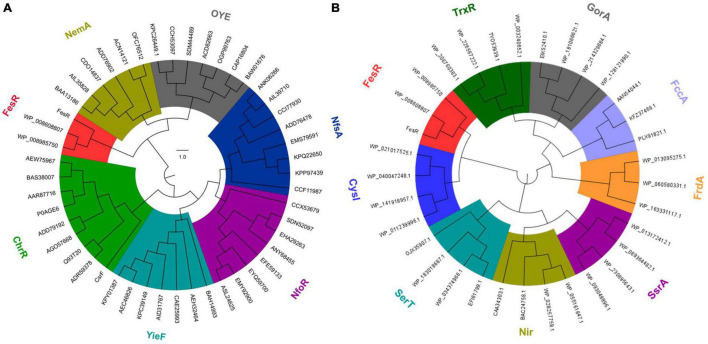
Evolutionary relationship of FesR with the reported aerobic chromate and selenite reductases. FesR and its similar proteins in other *Alishewanella* strains were separated from other chromates (NemA, OYE, NfsA, NfoR, YieF, and ChrR) reductase families **(A)** and selenite (TrxR, GorA, FccA, FrdA, SsrA, Nir, SerT and CysI) reductase families **(B)**.

To identify the function of FesR (AAY72_06850), the conserved domain of FesR was analyzed *in silico*. First, the amino acid sequence of FesR was blasted in the NCBI conserved domain database. The result showed FesR consisted of two conserved domains, including a FAD and two [4Fe-4S] cluster-binding sites. Second, the predicted FAD (16–520 aa) and the 4Fe-4S cluster (525–625 aa)-binding sequences were used to search in the HHpred server. Finally, the most similar sequences were chosen to align with the related sequences in FesR. The alignment result indicated that FesR shared nine identical residues with the reported FAD-binding proteins (Figure 1B) and 20 identical residues with the reported 4Fe-4S-binding proteins (Figure 1A). One of the identical residues, Arg385, was reported to associate with FAD binding in a flavin protein, ADPS (Razeto et al., 2007). Two 4Fe-4S-binding motifs, CXXCXXCXXXC (Gomes et al., 1999), were found in the FesR sequence (Figure 1B). Generally, the Fe-S cluster and FAD were associated with oxidoreduction reactions. In addition, no transmembrane motifs or signal peptides were predicted in the sequence of FesR. These results indicated that FesR might be an intracellular reductase. The amino acids of FesR were further aligned with the reported Cr(VI) and Se(IV) reductases. The phylogenetic analysis showed that FesR was separated from other aerobic Cr(VI) reductases (Figure 2A) and Se(IV) reductases (Figure 2B).

### *In vivo* Evidence of FesR Functioning as Cr(VI) and Se(IV) Reductases

**FIGURE 3:**
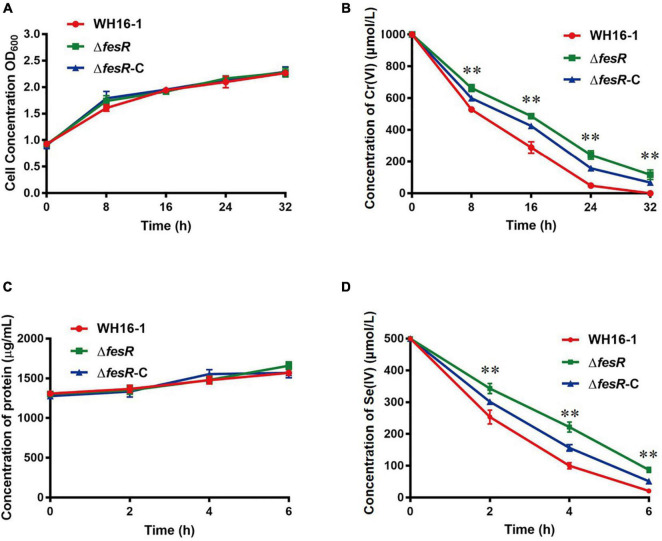
FesR is involved in the Cr(VI) and Se(IV) reduction in *Alishewanella* sp. WH16-1. Growth curves of WH16-1 (wild type), Δ*fesR* (mutant) and Δ*fesR*-C (complementation) strains in Luria-Bertani (LB) medium with 1000 μmol/L K_2_CrO_4_ **(A)** and 500 μmol/L Na_2_SeO_3_ **(C)**, respectively. Reduction curves of WH16-1, Δ*fesR* and Δ*fesR*-C in LB medium with 1000 μmol/L K_2_CrO_4_ **(B)** and 500 μmol/L Na_2_SeO_3_ **(D)**, respectively. The values represent averages and standard deviations (SD) of three replicates. ^**^stands for the statistically highly significant as *p* < 0.001 between the wild type and mutant strains.

To verify the function of FesR, the complementation strain Δ*fesR*-C was constructed based on the mutant strain Δ*fesR* (Supplementary Figure 1). Then, the Cr(VI) and Se(IV) reduction capacities of WH16-1, Δ*fesR*, and Δ*fesR*-C were tested under the same growth conditions (Figures 3A,C). The results showed that the mutant strain lost partial Cr(VI) and Se(IV) reduction capacities and that the complementation strain recovered partial reduction capacities (Figures 3B,D). WH16-1 reduced 100% 1000 μmol/L Cr(VI) (Figure 3B) and 95.8% 500 μmol/L Se(IV) (Figure 3D), while Δ*fesR* reduced 88.3% Cr(VI) (Figure 3B) and 82.5% Se(IV) (Figure 3D) at the same time. These results implied that FesR participated in Cr(IV) and Se(IV) reduction in strain WH16-1.

**FIGURE 4:**
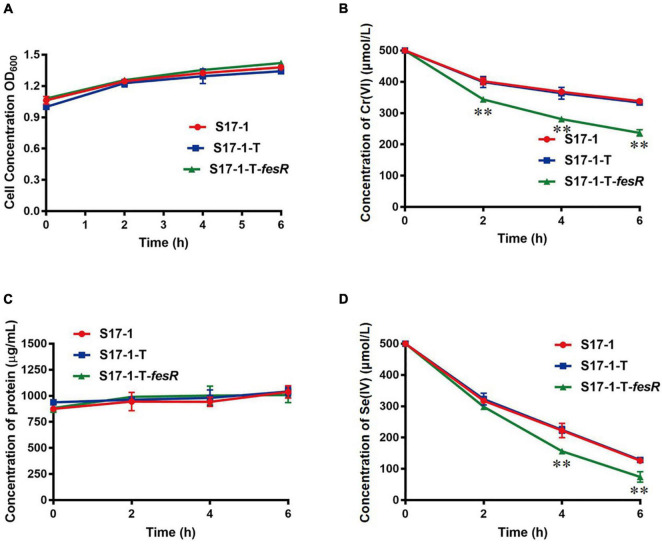
FesR enhanced the Cr(VI) and Se(IV) reduction capacities of *Escherichia coli* Growth curves of *E. coli* S17-1, *E. coli* S17-1-T and *E. coli* S17-1-T-*fesR* in LB broth with 500 μmol/L K_2_CrO_4_ **(A)** and Na_2_SeO_3_ **(C)**, respectively. Reduction curves of *E. coli* S17-1, *E. coli* S17-1-T and *E. coli* S17-1-T-*fesR* in Luria-Bertani (LB) broth with 500 μmol/L K_2_CrO_4_ **(B)** and Na_2_SeO_3_ **(D)**, respectively. Data were expressed as mean ± SD of the biological three replicates. ^**^stands for the statistically highly significant as *p* < 0.001 between the overexpressed strain *E. coli* S17-1-T-*fesR* and control strains (*E. coli* S17-1 and *E. coli* S17-1-T).

To gain further insight, FesR was overexpressed in *E. coli* S17-1. The Cr(VI) and Se(IV) reduction capacities of the overexpression strain S17-1-T-*fesR* were detected with *E. coli* S17-1 and *E. coli* S17-1-T (carrying the plasmid pGEM-T) as the control. The results showed that the Cr(VI) and Se(IV) reduction efficiencies of S17-1-T-*fesR* were increased 20.3% and 10.4% compared with S17-1 and S17-1-T at the same time, respectively (Figure 4). These results indicated that FesR enhanced the Cr(VI) and Se(IV) reduction capacities of *E. coli*.

### *In vitro* Evidence of FesR Functioning as Cr(VI) and Se(IV) Reductases

**FIGURE 5:**
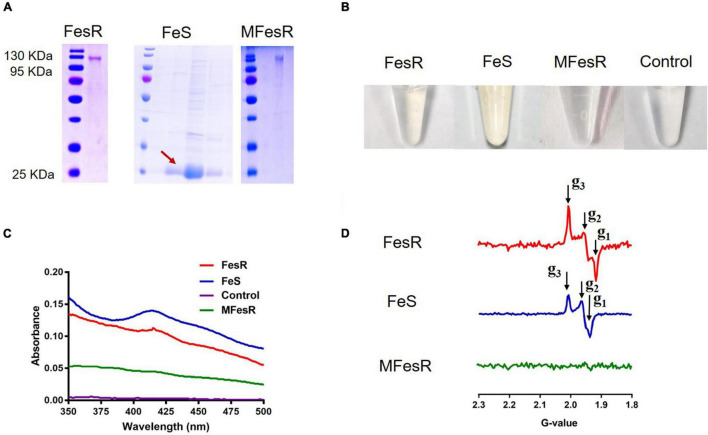
The characteristics of FesR. **(A)** The purified FesR, Fe-S (only contain the 4Fe-4S binding domains) and MFesR (4Fe-4S cluster binding domains were mutant) were analyzed by SDS-PAGE. **(B)** The purified FesR, Fe-S and MFesR. **(C)** The UV-VIS spectrums of FesR, Fe-S, MFesR and Tris-HCl buffer (pH 8.0). **(D)** The EPR spectrums of FesR, Fe-S and MFesR.

To identify the Cr(VI) and Se(IV) reduction capacities of FesR, GST-tagged FeS (only contain Fe-S domain), and mutant MFesR (4Fe-4S cluster binding domains were mutant) were purified by the purification method of FesR. SDS-PAGE (15%) gel was then used to analyze FesR, Fe-S, and MFesR (Figure 5A). FesR resolved as a single band between 130 and 95 kDa (Figure 5A), consistent with the predicted 127 kDa molecular weight. The purified FesR and FeS show a similar color, while no obvious color was observed in the purified MFesR (Figure 5B). This phenomenon was supported by the UV-VIS spectrum and EPR results. The UV-VIS spectrum result showed that FesR and FeS exhibited an absorption peak at 415nm (Figure 5C), which was similar to the Fe-S cluster spectrum of Rtel1 (Landry and Ding, 2014). The EPR spectrum of FesR (g_1_ = 1.91779, g_2_ = 1.95846, g_3_ = 2.00758) and FeS (g_1_ = 1.93772, g_2_ = 1.96635, g_3_ = 2.00718) were typical 4Fe-4S cluster spectrum (Figure 5D) (Ren et al., 2009; Landry and Ding, 2014).

**FIGURE 6:**
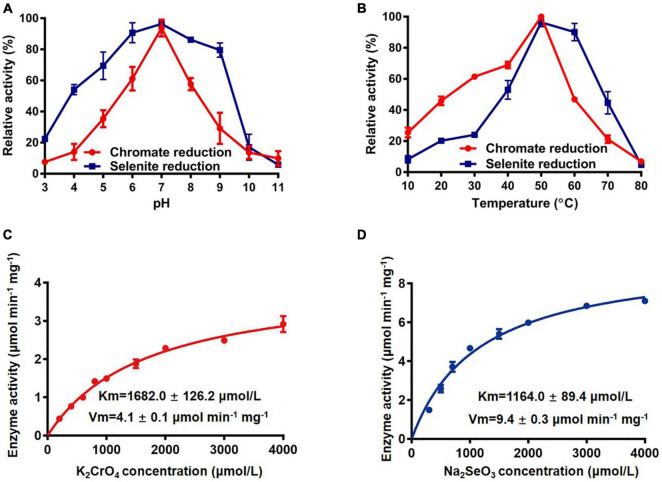
The enzymatic reaction rate of FesR. Effects of pH **(A)** and temperature **(B)** on the enzymatic activity. The Cr(VI) **(C)** and Se(IV) **(D)** reduction enzyme kinetics were determined at pH 7.0 and 50°C. Data are the means ± SD of three replicates.

Enzyme assays of FesR were then carried out. The activity of FesR required NADH or NADPH as an electron donor. There were no significant differences for FesR to use either NADH or NADPH as an electron donor (Supplementary Figure 2A). Using NADPH as an electron donor, the optimum pH and temperature of the FesR were investigated. The optimum pH and temperature for both Cr(VI) and Se(IV) reduction were 7 (Figure 6A) and 50°C (Figure 6B), respectively. The relative activity decreased at acid, alkaline, or high- or low-temperature conditions. The FesR has a preference to reduce Se(IV) where equimolar Cr(VI) and Se(IV) were added together, and this result was similar to the result of a separate experiment (Supplementary Figure 2B). The Km values for Cr(VI) and Se(IV) reduction were 1682.0 ± 126.2 μmol/L and 1164.0 ± 89.4 μmol/L, and the Vmax values for Cr(VI) and Se(IV) reduction were 4.1 ± 0.1 μmol min^–1^ mg^–1^ and 9.4 ± 0.3 μmol min^–1^ mg^–1^, respectively (Figures 6C,D). The Km and Vmax values of FesR showed that the FesR has higher affinity and reduction efficiency to Se(IV) than that to Cr(VI).

### The 4Fe-4S Cluster and Flavin Adenine Dinucleotide Are Essential to FesR

**FIGURE 7:**
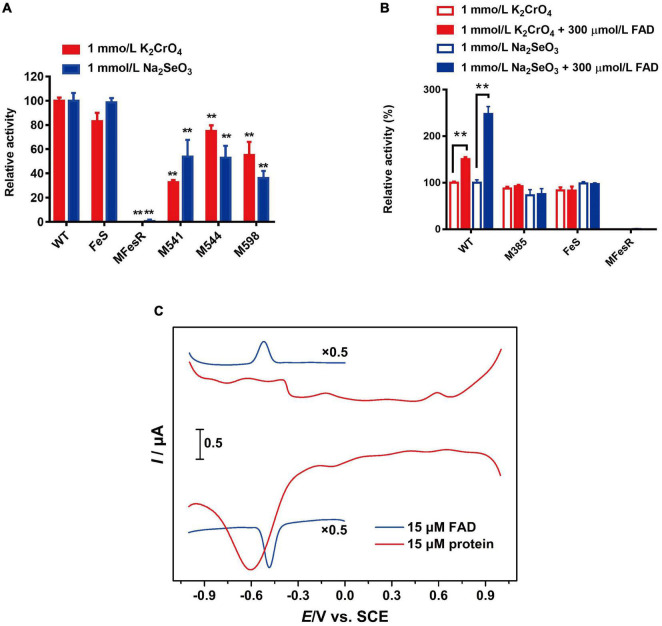
Function of co-factors and redox potential analysis. Effects of 4Fe-4S cluster **(A)** and flavin adenine dinucleotide (FAD) **(B)** on the FesR enzymatic activity. Samples were prepared in triplicate, and the results are presented as the means ± SD. DPVs of bare GC electrodes in 50 mM Tris-HCl buffer solution (pH = 8) with 15 μmol/L FesR or 0.1 mmol/L FAD **(C)**. Potential increment: 4 mV; amplitude: 50 mV; pulse width: 0.06 s; pulse period: 0.5 s. **stands for the statistically highly significant as *P* < 0.001.

The predicted 4Fe-4S cluster-binding residues Cys541, Cys544, Cys598, and all 8 Cys (Cys538, Cys541, Cys544, Cys548, Cys549, Cys595, Cys598, Cys601, and Cys605) were mutated to confirm the role of the 4Fe-4S cluster in FesR. Both the Cr(VI) and Se(IV) reduction capacities of M541, M544, M598, and MFesR were notably decreased (Figure 7A). These findings indicate that these 8 cysteines were involved in the 4Fe-4S cluster binding and that the 4Fe-4S cluster was essential to the enzyme activity of FesR.

The importance of FAD to FesR was also evaluated. The Cr(VI) and Se(IV) reduction capacities of FesR were tested with additional FAD since no FAD was bound to purified FesR, as described above. The results demonstrated that the Cr(VI) and Se(IV) reduction capacities were both increased when an additional 300 μmol/L FAD was added (Figure 7B). Furthermore, FAD couldn’t enhance the enzyme activity of FesR when the predicted FAD-binding residue Arg385 was mutated and the absence of the FAD binding domain (Figure 7B). These findings suggested that electrons might be transferred from FesR to FAD, and the resulting FADH_2_ subsequently reduced Cr(VI) and Se(IV). This hypothesis was also verified by the fact that the reduction potential of FesR (*Ep* = -0.6 V v*s.* SCE) was low enough to reduce oxidative FAD (*Ep* = -0.52 V *vs.* SCE) (Figure 7C).

## Discussion

FesR was determined to associate with Cr(VI) detoxification in *Alishewanella* sp. WH16-1 by Tn*5* transposon mutagenesis (Xia et al., 2018a). In this study, we verified the Cr(VI) and Se(IV) reduction capacities of FesR. According to the mutation, overexpression, and *in vitro* enzyme activity tests, FesR exhibited noticeable Cr(VI) and Se(IV) reduction capacities. FesR showed significant differences with the reported Cr(VI) and Se(IV) reductases in several aspects: (i) The FesR amino acids sequences, separated from other Cr(VI) and Se(IV) reductases, belong to a novel and independent branch (Figure 2). (ii) The FesR contains two copies of [4Fe-4S] clusters and FAD-binding conserved domains, both of which play an important role in the activity of FesR (Supplementary Table 3). (iii) The kinetic parameters (Km and Vmax) of FesR are different from those of the reported Cr(VI) and Se(IV) reductases (Supplementary Table 3).

Interestingly, no FAD was detected on FesR based on the UV-VIS spectrum, but the additional FAD could enhance the Cr(VI) and Se(IV) reduction capacities of FesR. According to the site-directed mutagenesis and redox potential results, the electron transfer was taken from the electron donor (NADPH/NADH) to FesR [two (4Fe-4S) cluster centers]. Then, the reduced FesR transferred electrons to Cr(VI)/Se(IV) or FAD (Figure 7). The generated FADH_2_ might be bound to FesR to reduce Cr(VI)/Se(IV) as well as GorA (Wang et al., 2019) or separated from the enzyme to reduce Cr(VI)/Se(IV) directly (Cervantes et al., 2001). In this process, FAD enhanced the electron transfer efficiency. FAD likewise acts as an extracellular electron shuttle to enhance the reduction capacity in *Geobacter* and *Shewanella* (Yang et al., 2012; Kotloski and Gralnick, 2013). The difference is that FAD accepts electrons from FesR and transfers the electrons to the terminal electron acceptor [Cr(VI) and Se(IV)] under aerobic condition, while the FAD is hydrolyzed to FMN by UshA and then the generated FMN participates in electron transport from Mtr pathway to the terminal electron acceptor under anaerobic condition (Yang et al., 2012). These findings suggested that FAD is involved in electron transport in different ways under aerobic or anaerobic conditions and the reduction mechanism of FesR is different from the reported Cr(VI)/Se(IV) reductases.

According to the previous and above research, *Alishewanella* sp. WH16-1 have evolved multiple mechanisms to cope with Cr(VI), as follows: (i) exporting the intracellular Cr(VI) to the extracellular environment by ChrA (Xia et al., 2016, 2018a), (ii) reducing highly toxic Cr(VI) to Cr(III) by FesR and CsrF (Xia et al., 2018b), (iii) reducing cellular oxidative stress by ChrC (Xia et al., 2016) and Cytbd (Xia et al., 2018a), and (iv) repairing DNA damage by RuvRCAB (Wu et al., 2019). In addition, a TonB-dependent receptor was found to contribute to the Cr(VI) resistance by Tn*5* transposon mutagenesis (Xia et al., 2018a). TonB-dependent receptors were upregulated in the transcriptome results of *Caulobacter crescentus* (Hu et al., 2005) and the proteome results of *Shewanella oneidensis* (Brown et al., 2006) under Cr(VI) stress. The TonB-dependent receptor was involved in iron uptake (Viti et al., 2014), and iron plays an important role in the active center of the Cr(VI) reductase FesR and the Cr(VI) resistance protein Cytbd (Xia et al., 2018a). Accordingly, iron metabolism might also be involved in the Cr(VI) detoxification, which requires further study.

The *Alishewanella* sp. WH16-1, can remove multiple pollutants, has been used to treat lead and cadmium contaminated paddy soil (Zhou et al., 2016; Shi et al., 2018; Yu et al., 2022). The *Alishewanella* sp. WH16-1 also exhibits a good prospect of application in the Se(IV) and Cr(VI) remediation, because of the excellent Cr(VI) and Se(IV) reduction abilities. Moreover, the products of the reduction of Se(IV) and Cr(VI) can be extracted from *Alishewanella* sp. WH16-1 in format at Se(0)- and Cr(III)-nanoparticles, respectively (Xia et al., 2018b). Many different sources Se(0)- and Cr(III)-nanoparticles have been widely used in medicine, feeding, and environmental remediation (Wadhwani et al., 2016; Kanakalakshmi et al., 2017; Satgurunathan et al., 2019).

## Data Availability Statement

The original contributions presented in the study are included in the article/Supplementary Material, further inquiries can be directed to the corresponding author/s.

## Author Contributions

GW and XX designed this study. ZZ, XX, LZ, YD, and LY performed the experiments. XX wrote the original manuscript. ZZ, GW, and SZ edited the manuscript. All authors contributed to the article and approved the submitted version.

## Conflict of Interest

The authors declare that the research was conducted in the absence of any commercial or financial relationships that could be construed as a potential conflict of interest.

## Publisher’s Note

All claims expressed in this article are solely those of the authors and do not necessarily represent those of their affiliated organizations, or those of the publisher, the editors and the reviewers. Any product that may be evaluated in this article, or claim that may be made by its manufacturer, is not guaranteed or endorsed by the publisher.

## Footnotes

1
http://tree.bio.ed.ac.uk/software/figtree/

## References

[B1] Ackerley D. F. Gonzalez C. F. Park C. H. Blake R. Keyhan M. Matin A. (2004b). Chromate-reducing properties of soluble flavoproteins from *Pseudomonas putida* and *Escherichia coli*. *Appl. Environ. Microbiol.* 70 873–882. 10.1128/aem.70.2.873-882.2004 14766567 PMC348923

[B2] Ackerley D. F. Gonzalez C. F. Keyhan M. Blake R. Matin A. (2004a). Mechanism of chromate reduction by the *Escherichia coli* protein, NfsA, and the role of different chromate reductases in minimizing oxidative stress during chromate reduction. *Environ. Microbiol.* 6 851–860. 10.1111/j.1462-2920.2004.00639.x 15250887

[B3] Almagro Armenteros J. J. Tsirigos K. D. Sønderby C. K. Petersen T. N. Winther O. Brunak S. (2019). SignalP 5.0 improves signal peptide predictions using deep neural networks. *Nat. Biotechnol.* 37 420–423. 10.1038/s41587-019-0036-z 30778233

[B4] Alva V. Nam S. Z. Soding J. Lupas A. N. (2016). The MPI bioinformatics Toolkit as an integrative platform for advanced protein sequence and structure analysis. *Nucleic Acids Res.* 44 W410–W415. 10.1093/nar/gkw348 27131380 PMC4987908

[B5] Belchik S. M. Kennedy D. W. Dohnalkova A. C. Wang Y. Sevinc P. C. Wu H. (2011). Extracellular reduction of hexavalent chromium by cytochromes MtrC and OmcA of *Shewanella oneidensis* MR-1. *Appl. Environ. Microbiol.* 77 4035–4041. 10.1128/AEM.02463-10 21498755 PMC3131624

[B6] Brown S. D. Thompson M. R. Verberkmoes N. C. Chourey K. Shah M. Zhou J. (2006). Molecular dynamics of the Shewanella oneidensis response to chromate stress. *Mol. Cell. Proteomics* 5 1054–1071. 10.1074/mcp.M500394-MCP200 16524964

[B7] Cervantes C. Campos-Garcia J. Devars S. Gutierrez-Corona F. Loza-Tavera H. Torres-Guzman J. C. (2001). Interactions of chromium with microorganisms and plants. *FEMS Microbiol. Rev.* 25 335–347. 11348688 10.1111/j.1574-6976.2001.tb00581.x

[B8] Glasser N. R. Saunders S. H. Newman D. K. (2017). The colorful world of extracellular electron shuttles. *Annu. Rev. Microbiol.* 71 731–751. 10.1146/annurev-micro-090816-093913 28731847 PMC5679407

[B9] Gomes C. M. Lemos R. S. Teixeira M. Kletzin A. Huber H. Stetter K. O. (1999). The unusual iron sulfur composition of the *Acidianus ambivalens* succinate dehydrogenase complex. *Biochim. Biophys. Acta Bioenerg.* 1411 134–141. 10.1016/S0005-2728(99)00046-8 10216159

[B10] Han H. Zheng Y. Zhou T. Liu P. Li X. (2021). Cu(II) nonspecifically binding chromate reductase NfoR promotes Cr(VI) reduction. *Environ. Microbiol.* 23 415–430. 10.1111/1462-2920.15329 33201569

[B11] Hu P. Brodie E. L. Suzuki Y. McAdams H. H. Andersen G. L. (2005). Whole-genome transcriptional analysis of heavy metal stresses in *Caulobacter crescentus*. *J. Bacteriol.* 187 8437–8449. 10.1128/JB.187.24.8437-8449.2005 16321948 PMC1317002

[B12] Jobby R. Jha P. Yadav A. K. Desai N. (2018). Biosorption and biotransformation of hexavalent chromium [Cr(VI)]: a comprehensive review. *Chemosphere* 207 255–266. 10.1016/j.chemosphere.2018.05.050 29803157

[B13] Joosten V. van Berkel W. J. (2007). Flavoenzymes. *Curr. Opin. Chem. Biol.* 11 195–202. 10.1016/j.cbpa.2007.01.010 17275397

[B14] Kanakalakshmi A. Janaki V. Shanthi K. Kamala-Kannan S. (2017). Biosynthesis of Cr(III) nanoparticles from electroplating wastewater using chromium-resistant *Bacillus subtilis* and its cytotoxicity and antibacterial activity. *Artif. Cells Nanomed. Biotechnol*. 45 1304–1309. 10.1080/21691401.2016.1228660 27608920

[B15] Kotloski N. J. Gralnick J. A. (2013). Flavin electron shuttles dominate extracellular electron transfer by *Shewanella oneidensis*. *mBio* 4:e00553-12. 10.1128/mBio.00553-12 23322638 PMC3551548

[B16] Krogh A. Larsson B. von Heijne G. Sonnhammer E. L. L. (2001). Predicting transmembrane protein topology with a hidden markov model: application to complete genomes. *J. Mol. Biol.* 305 567–580. 10.1006/jmbi.2000.4315 11152613

[B17] Kumar S. Stecher G. Tamura K. (2016). MEGA7: molecular evolutionary genetics analysis version 7.0 for bigger datasets. *Mol. Biol. Evol.* 33 1870–1874. 10.1093/molbev/msw054 27004904 PMC8210823

[B18] Landry A. P. Ding H. (2014). The N-terminal domain of human DNA helicase Rtel1 contains a redox active iron-sulfur cluster. *Biomed Res. Int.* 2014:285791. 10.1155/2014/285791 25147792 PMC4131540

[B19] Lunk H.-J. (2015). Discovery, properties and applications of chromium and its compounds. *ChemTexts* 1:6. 10.1007/s40828-015-0007-z

[B20] Moukarzel A. (2009). Chromium in parenteral nutrition: Too little or too much? *Gastroenterology* 137 (5 Suppl.), S18–S28. 10.1053/j.gastro.2009.08.048 19874946

[B21] Opperman D. J. Piater L. A. van Heerden E. (2008). A novel chromate reductase from *Thermus scotoductus* SA-01 related to old yellow enzyme. *J. Bacteriol.* 190 3076–3082. 10.1128/JB.01766-07 18263719 PMC2293266

[B22] Rayman M. P. (2012). Selenium and human health. *Lancet* 379 1256–1268. 10.1016/s0140-6736(11)61452-9 22381456

[B23] Razeto A. Mattiroli F. Carpanelli E. Aliverti A. Pandini V. Coda A. (2007). The crucial step in ether phospholipid biosynthesis: structural basis of a noncanonical reaction associated with a peroxisomal disorder. *Structure* 15 683–692. 10.1016/j.str.2007.04.009 17562315

[B24] Ren B. Duan X. Ding H. (2009). Redox control of the DNA damage-inducible protein DinG helicase activity via its iron-sulfur cluster. *J. Biol. Chem.* 284 4829–4835. 10.1074/jbc.M807943200 19074432 PMC2643519

[B25] Robert X. Gouet P. (2014). Deciphering key features in protein structures with the new ENDscript server. *Nucleic Acids Res.* 42 W320–W324. 10.1093/nar/gku316 24753421 PMC4086106

[B26] Robins K. J. Hooks D. O. Rehm B. H. Ackerley D. F. (2013). *Escherichia coli* NemA is an efficient chromate reductase that can be biologically immobilized to provide a cell free system for remediation of hexavalent chromium. *PLoS One* 8:e59200. 10.1371/journal.pone.0059200 23527133 PMC3596305

[B27] Satgurunathan T. Bhavan P. S. Joy R. (2019). Green synthesis of chromium nanoparticles and their effects on the growth of the prawn *Macrobrachium rosenbergii* Post-larvae. *Biol. Trace Elem. Res*. 187 543–552. 10.1007/s12011-018-1407-x 29948910

[B28] Scopes R. K. (2002). “Enzyme activity and assays,” in *Encyclopedia of Life Sciences*, (Hoboken, NJ: John Wiley & Sons, Inc), 1–6. 10.1038/npg.els.0000712

[B29] Shi X. Zhou G. Liao S. Shan S. Wang G. Guo Z. (2018). Immobilization of cadmium by immobilized *Alishewanella* sp. WH16-1 with alginate-lotus seed pods in pot experiments of Cd-contaminated paddy soil. *J. Hazard. Mater.* 357 431–439. 10.1016/j.jhazmat.2018.06.027 29929096

[B30] Tan Y. Wang Y. Wang Y. Xu D. Huang Y. Wang D. (2018). Novel mechanisms of selenate and selenite reduction in the obligate aerobic bacterium *Comamonas testosteroni* S44. *J. Hazard. Mater.* 359 129–138. 10.1016/j.jhazmat.2018.07.014 30014908

[B31] Thatoi H. Das S. Mishra J. Rath B. P. Das N. (2014). Bacterial chromate reductase, a potential enzyme for bioremediation of hexavalent chromium: a review. *J. Environ. Manage.* 146 383–399. 10.1016/j.jenvman.2014.07.014 25199606

[B32] Thompson J. D. Higgins D. G. Gibson T. J. (1994). CLUSTAL W: improving the sensitivity of progressive multiple sequence alignment through sequence weighting, position-specific gap penalties and weight matrix choice. *Nucleic Acids Res.* 22 4673–4680. 7984417 10.1093/nar/22.22.4673 PMC308517

[B33] Viti C. Marchi E. Decorosi F. Giovannetti L. (2014). Molecular mechanisms of Cr(VI) resistance in bacteria and fungi. *FEMS Microbiol. Rev.* 38 633–659. 10.1111/1574-6976.12051 24188101

[B34] Wadhwani S. A. Shedbalkar U. U. Singh R. Chopade B. A. (2016). Biogenic selenium nanoparticles: current status and future prospects. *Appl. Microbiol. Biotechnol*. 100 2555–2566. 10.1007/s00253-016-7300-7 26801915

[B35] Wang D. Rensing C. Zheng S. (2022). Microbial reduction and resistance to selenium: mechanisms, applications and prospects. *J. Hazard. Mater.* 421:126684. 10.1016/j.jhazmat.2021.126684 34339989

[B36] Wang D. Xia X. Wu S. Zheng S. Wang G. (2019). The essentialness of glutathione reductase GorA for biosynthesis of Se(0)-nanoparticles and GSH for CdSe quantum dot formation in *Pseudomonas stutzeri* TS44. *J. Hazard. Mater.* 366 301–310. 10.1016/j.jhazmat.2018.11.092 30530022

[B37] Wang Y. Su H. Gu Y. Song X. Zhao J. (2017). Carcinogenicity of chromium and chemoprevention: a brief update. *Onco Targets Ther.* 10 4065–4079. 10.2147/OTT.S139262 28860815 PMC5565385

[B38] Wu S. Xia X. Wang D. Zhou Z. Wang G. (2019). Gene function and expression regulation of RuvRCAB in bacterial Cr(VI), As(III), Sb(III), and Cd(II) resistance. *Appl. Microbiol. Biotechnol.* 103 2701–2713. 10.1007/s00253-019-09666-6 30729256

[B39] Xia X. Li J. Liao S. Zhou G. Wang H. Li L. (2016). Draft genomic sequence of a chromate- and sulfate-reducing *Alishewanella* strain with the ability to bioremediate Cr and Cd contamination. *Stand. Genomic Sci.* 11:48. 10.1186/s40793-016-0169-3 27499827 PMC4974768

[B40] Xia X. Wu S. Li L. Xu B. Wang G. (2018a). The Cytochrome bd complex is essential for chromate and sulfide resistance and is regulated by a GbsR-type regulator, CydE, in *Alishewanella* Sp. WH16-1. *Front. Microbiol.* 9:1849. 10.3389/fmicb.2018.01849 30147685 PMC6096048

[B41] Xia X. Wu S. Li N. Wang D. Zheng S. Wang G. (2018b). Novel bacterial selenite reductase CsrF responsible for Se(IV) and Cr(VI) reduction that produces nanoparticles in *Alishewanella* sp. WH16-1. *J. Hazard. Mater.* 342 499–509. 10.1016/j.jhazmat.2017.08.051 28881274

[B42] Xia X. Wu S. Zhou Z. Wang G. (2021). Microbial Cd(II) and Cr(VI) resistance mechanisms and application in bioremediation. *J. Hazard. Mater.* 401:123685. 10.1016/j.jhazmat.2020.123685 33113721

[B43] Yang Y. Xu M. Guo J. Sun G. (2012). Bacterial extracellular electron transfer in bioelectrochemical systems. *Process Biochem.* 47 1707–1714. 10.1016/j.procbio.2012.07.032

[B44] Yu Y. Shi K. Li X. Luo X. Wang M. Li L. (2022). Reducing cadmium in rice using metallothionein surface-engineered bacteria WH16-1-MT. *Environ. Res*. 203:111801. 10.1016/j.envres.2021.111801 34339701

[B45] Zhou G. Xia X. Wang H. Li L. Wang G. Zheng S. (2016). Immobilization of lead by *Alishewanella* sp. WH16-1 in pot experiments of Pb-contaminated paddy soil. *Water Air Soil Pollut*. 227:339. 10.1007/s11270-016-3040-7

