# Supplementary material for: Identification of a Novel Chromate and Selenite Reductase FesR in Alishewanella sp. WH16-1
Source: Front Microbiol. 2022 Mar 8;13:834293. doi: 10.3389/fmicb.2022.834293 (PMC8957926; doi:10.3389/fmicb.2022.834293)
Supplement: Supplementary file 1 [file Data_Sheet_1.docx]

**Supplemental Material**

**Table S1 Bacterial strains and plasmids used in this study**

| **Strains or plasmids** | **Relevant properties or derivation** | **Source or reference** |
| --- | --- | --- |
| **Bacterial strains**  ***Alishewanella* sp.** |  |  |
| WH16-1 | Rif^r^; Km^s^, Tc^s^, Cm^s^ | (Zhou et al., 2016) |
| WH16-1-Δ*fesR* | *fesR* gene replaced by Tn*5*; Rif^r^, Km^r^ | (Chen et al., 2015) |
| WH16-1-Δ*fesR* -C | WH16-1-Δ*fesR* complemented with the *fesR* gene cloned in pCT-Zori vector; Rif^r^, Km^r^, Cm^r^ | This study |
| ***Escherichia coli*** |  |  |
| S17-1(λ*pir*) | *recA hsdR thi pro SmR RP4-2-TcR:: Mu-Km:: Tn7* | (Chen et al., 2015) |
| BL21 | B F^-^ *dcm ompT hsdS*(r_B_^-^m_B_^-^) *gal* λ(DE3) | Novagen |
|  |  |  |
| **Plasmids** |  |  |
| pCT-Zori | Cm^r^ | (Chen et al., 2015) |
| pCT-Zori- *fesR* | pCT-Zori vector cloned with the *fesR* gene, Cm^r^ | This study |
| pGEM-T  pGEM-T-*fesR* | Amp^r^  pGEM-T vector cloned with the *fesR* gene, Cm^r^ | Promega  This study |
| pGEX-6P-1 | His6 Tag expression vector, Km^r^ | Novagen |
| pGEX-6P-1- *fesR* | *fesR* in frame fusion to the multiple sites of pGEX-6P-1, Km^r^ | This study |
| pGEX-6P-1-*m fesR* | *fesR* in frame fusion to the multiple sites of pGEX-6P-1, Km^r^, the nucleotide acids of cysteine (Cys538, Cys541, Cys544, Cys548, Cys549, Cys595, Cys598, Cys601 and Cys605) were replaced by the nucleotide acids of alanine | This study |

**Table S2 Primers used in this study**

| Primer | Sequence (5’-3’) | Description |
| --- | --- | --- |
| C-*fesR*F | AAAAAGCTTGTTAATGCCGGGCGTTTT | For complementation of Δ*fesR* |
| C-*fesR*R | AAA GAGCTCGTACCGTAATTAGTCACTTTGTG |  |
| V- *fesR*F | TAATGGTGGGATCAGAAGG | For verification the *fesR* mutant and complemented strain |
| V*-fesR*R | ATCCAGCGTTATGCCG |  |
| pGEX*-fesR*F | AAAGAATTCATGCAGCCGCAGTTTCT | For FesR protein overexpression |
| pGEX*-fesR*R | AAA CTCGAG GTACCGTAATTAGTCACTTTGTG |  |
| Arg385F | CGGCGCGGTAGCAGAAACCGGCA | For site-directed mutagenesis of Arg385 |
| Arg385R | TGCTACCGCGCCGACCGCCGGAA |  |
| Cys541F | ATGTATTGAAGCAGGCTTTTGTG | For site-directed mutagenesis of Cys541 |
| Cys541R | TGCTTCAATACATTTATCGACCA |  |
| Cys544F | ATGTGGCTTTGCAGAGCCAGTTT | For site-directed mutagenesis of Cys544 |
| Cys544R | TGCAAAGCCACATTCAATACATT |  |
| Cys598F | CTGCGCCGCCGCAGGCCTGTGTA | For site-directed mutagenesis of Cys598 |
| Cys598R | TGCGGCGGCGCAGGTATCAGTGC |  |

* The underlined sequence denotes the restriction enzyme sites.

**Table S3 Comparation of FesR with other Cr(VI) and Se(IV) reductases**

| **Reductase** | **Cofactors** | **Km (μmol/L)** | **Vmax (μmol min^-1^ mg^-1^)** | **Reference** |
| --- | --- | --- | --- | --- |
| **Cr(VI) reduction** | | | | |
| ChrR | FMN | 260 | 8.8 | (Ackerley *et al*., 2004b) |
| YieF | FMN | 200 | 5 | (Ackerley *et al*., 2004b) |
| NemA | FMN | 212.1 | 0.95 | (Shi *et al*., 2021) |
| NfoR | FMN | 421.5 | 52.08 | (Han *et al*., 2021) |
| OYE family | FMN | 8.4 | 16 | (Opperman *et al*., 2008; Hunter 2013) |
| NfsA | FMN | 36 | 0.25 | (Ackerley *et al*., 2004a) |
| **Se(IV) reduction** | | | | |
| SerT | Molybdenum | 180 | 0.061 | (Tan *et al*., 2018) |
| CysI | Siroheme,  Fe_4_-S_4_ cluster | - | - | (Tan *et al*., 2018; Ostrowski *et al*., 1989) |
| TrxR | FAD | 2910 | 11.2 | (Yasir *et al*., 2020) |
| GorA | FAD | 4999 | 3.947 | (Wang *et al*., 2018) |
| SrrA | 4Fe-4S cluster | 145 | 23 | (Wells *et al*., 2019) |
| **Cr(VI) and Se(IV) reduction** | | | | |
| CsrF | FMN | 250.6 (Cr(VI))  204.1 (Se(IV)) | 11.92 (Cr(VI))  1.54 (Se(IV)) | (Xia *et al*., 2018b) |
| **FesR** | **FMN,**  **4Fe-4S cluster** | **1682 (Cr(VI))**  **1164 (Se(IV))** | **4.061 (Cr(VI))**  **9.427 (Se(IV))** | **This study** |


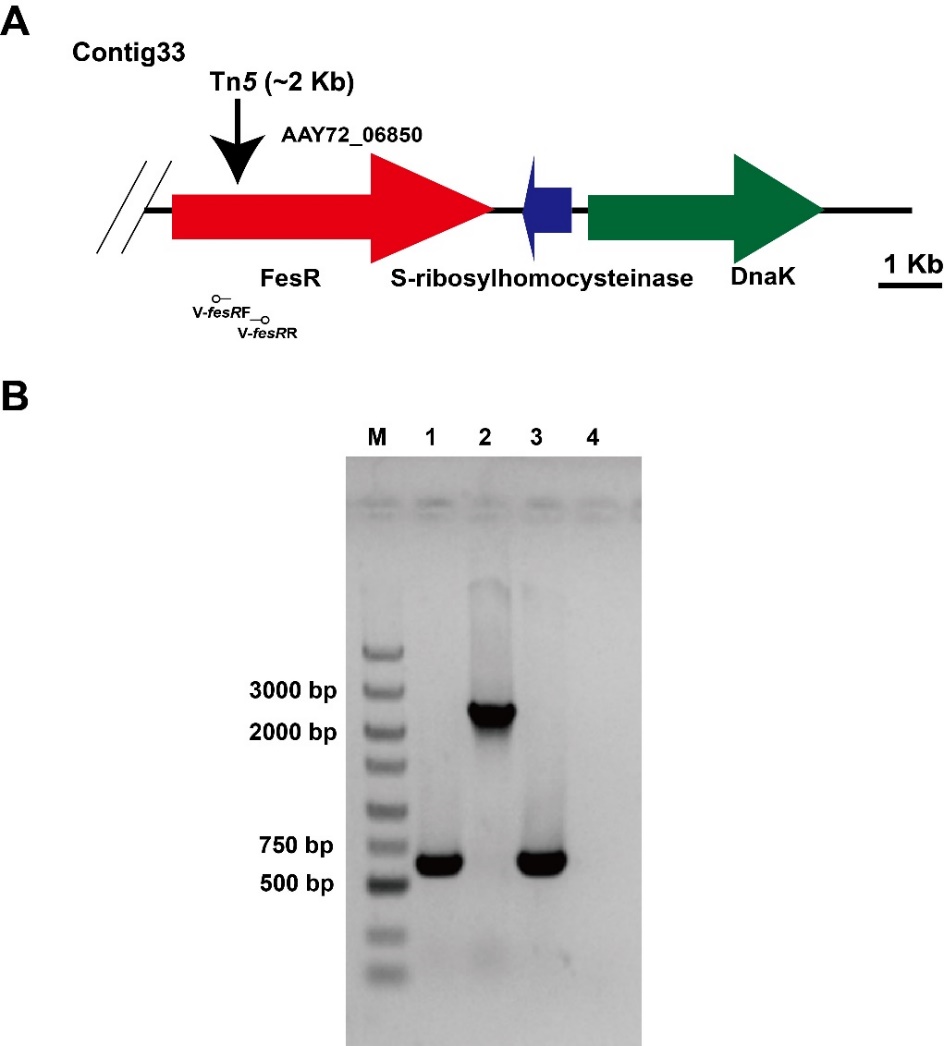


**FIG S1** The physical map of *fesR* in the genome of *Alishewanella* sp. WH16-1 and mutation and complementation analysis. (A) The genes were adjacent to *fesR*. (B) Mutation and complementation were verified by PCR. Lanes 1-3 are the PCR products of wild-type, mutant (Δ*fesR*) and complemented (Δ*fesR*-C) strains using the primers V-*fesR*F/V-*fesR*R, respectively. Lane 4 is a negative control using ddH_2_O as the template.


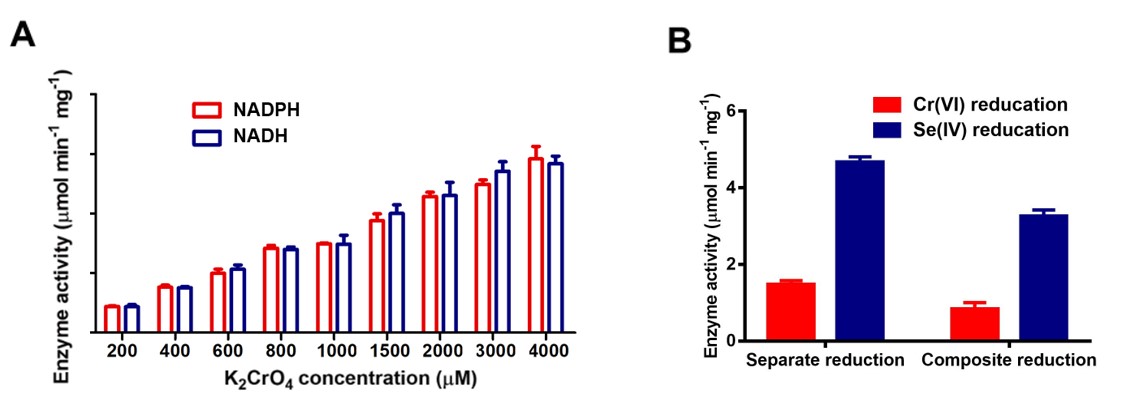


**FIG S2** Some enzyme assays of FesR. (A) Effects of NADPH and NADH on the enzyme activity of FesR. Different concentrations of K_2_CrO_4_ were reduced by FesR with different electron donor (NADPH or NADH). Data are the means ± SD of three replicates. (B) The preference of FesR to reduce Cr(VI) and Se(IV). In the separated or composite condition, 1mmol/L K_2_CrO_4_ and Na_2_SeO_3_ were reduced by FesR. Data are the means ± SD of three replicates.

**References**

Ackerley, D.F., Gonzalez, C.F., Keyhan, M., Blake, R., and Matin, A. (2004a) Mechanism of chromate reduction by the *Escherichia coli* protein, NfsA, and the role of different chromate reductases in minimizing oxidative stress during chromate reduction. *Environ Microbiol.* 6(8), 851-860. doi: 10.1111/j.1462-2920.2004.00639.x.

Ackerley, D.F., Gonzalez, C.F., Park, C.H., Blake, R., Keyhan, M., and Matin, A. (2004b). Chromate-reducing properties of soluble flavoproteins from *Pseudomonas putida* and *Escherichia coli*. *Appl Environ Microbiol.* 70(2), 873-882. doi:10.1128/aem.70.2.873-882.2004.

Han, H., Zheng, Y., Zhou, T., Liu, P., and Li, X. (2021) Cu(II) nonspecifically binding chromate reductase NfoR promotes Cr(VI) reduction. *Environ Microbiol.* 23(1), 415-430. doi: 10.1111/1462-2920.15329.

Hunter, W. J. (2013). A Rhizobium selenitireducens Protein Showing Selenite Reductase Activity. Current Microbiology, 68(3), 311–316. doi:10.1007/s00284-013-0474-7

Opperman, D.J., Piater, L.A., and van Heerden, E. (2008) A novel chromate reductase from *Thermus scotoductus* SA-01 related to old yellow enzyme. *J Bacteriol.* 190(8), 3076-3082. doi: 10.1128/JB.01766-07.

Ostrowski J, Wu JY, Rueger DC, Miller BE, Siegel LM, Kredich NM. (1989) Characterization of the cysJIH regions of Salmonella typhimurium and *Escherichia coli* B. DNA sequences of cysI and cysH and a model for the siroheme-Fe4S4 active center of sulfite reductase hemoprotein based on amino acid homology with spinach nitrite reductase. *J Biol Chem*. 264(26),15726-37. PMID: 2670946.

Shi, K., Radhakrishnan, M., Dai, X., Rosen, B. P., & Wang, G. (2021). NemA Catalyzes Trivalent Organoarsenical Oxidation and Is Regulated by the Trivalent Organoarsenical-Selective Transcriptional Repressor NemR. *Environ. Sci. Technol*. 55(9), 6485–6494. doi:10.1021/acs.est.1c00574.

Tan, Y., Wang, Y., Wang, Y., Xu, D., Huang, Y., Wang, D. et al. (2018) Novel mechanisms of selenate and selenite reduction in the obligate aerobic bacterium *Comamonas testosteroni* S44. *J Hazard Mater.* 359, 129-138. doi: 10.1016/j.jhazmat.2018.07.014.

Wang, D., Xia, X., Wu, S., Zheng, S., & Wang, G. (2018). The Essentialness of Glutathione Reductase GorA for Biosynthesis of Se(0)-nanoparticles and GSH for CdSe Quantum Dot Formation in *Pseudomonas stutzeri* TS44. *J Hazard Mater*. 366, 301-310. doi:10.1016/j.jhazmat.2018.11.092

Xia, X., Wu, S., Li, N., Wang, D., Zheng, S., and Wang, G. (2018) Novel bacterial selenite reductase CsrF responsible for Se(IV) and Cr(VI) reduction that produces nanoparticles in *Alishewanella* sp. WH16-1. *J Hazard Mater.* 342, 499-509. doi 10.1016/j.jhazmat.2017.08.051.

Yasir, M., Zhang, Y., Xu, Z., Luo, M., & Wang, G. (2020). NAD(P)H dependent thioredoxin-disulfide reductase TrxR is essential for tellurite and selenite reduction and resistance in *Bacillus* sp. Y3. *FEMS Microbiol Ecol*. 96(9), fiaa126. doi:10.1093/femsec/fiaa126
